# White versus gray matter: fMRI hemodynamic responses show similar characteristics, but differ in peak amplitude

**DOI:** 10.1186/1471-2202-13-91

**Published:** 2012-08-01

**Authors:** Leanne M Fraser, M Tynan Stevens, Steven D Beyea, Ryan C N D’Arcy

**Affiliations:** 1 Institute for Biodiagnostics (Atlantic), National Research Council, Halifax, NS, B3H 3A7, Canada; 2 Department of Psychology/Neuroscience, Dalhousie University, Halifax, Canada; 3 Department of Physics and Atmospheric Sciences, Dalhousie University, Halifax, Canada; 4 Department of Radiology, Dalhousie University, Halifax, Canada; 5 School of Biomedical Engineering, Dalhousie University, Halifax, Canada; 6 Department of Anatomy and Neurobiology, Dalhousie University, Halifax, Canada

**Keywords:** White matter, Functional connectivity, BOLD response, Hemodynamic response function, Event-related fMRI, Interhemispheric transfer

## Abstract

**Background:**

There is growing evidence for the idea of fMRI activation in white matter. In the current study, we compared hemodynamic response functions (HRF) in white matter and gray matter using 4 T fMRI. White matter fMRI activation was elicited in the isthmus of the corpus callosum at both the group and individual levels (using an established interhemispheric transfer task). Callosal HRFs were compared to HRFs from cingulate and parietal activation.

**Results:**

Examination of the raw HRF revealed similar overall response characteristics. Finite impulse response modeling confirmed that the WM HRF characteristics were comparable to those of the GM HRF, but had significantly decreased peak response amplitudes.

**Conclusions:**

Overall, the results matched *a priori* expectations of smaller HRF responses in white matter due to the relative drop in cerebral blood flow (CBF) and cerebral blood volume (CBV). Importantly, the findings demonstrate that despite lower CBF and CBV, white matter fMRI activation remained within detectable ranges at 4 T.

## Background

Historically, functional magnetic resonance imaging (fMRI) has focused on gray matter (GM). GM, however, only accounts for approximately 50% of total brain matter [1]. White matter (WM) activation in fMRI has been controversial. The idea that WM fMRI activation is related to underlying neural activation is counter to two main observations: 1) cerebral blood flow (CBF) and cerebral blood volume (CBV) are typically reduced in WM relative to GM [2-4], therefore the relatively small BOLD contrast signal is thought to be below the threshold of detection in WM; and 2) the source of the fMRI signal has been associated largely with post-synaptic potentials, the majority of which occur in GM [5]. Consequently, fMRI activation in WM is commonly dismissed as artifact.

Despite these preconceptions, a growing number of studies from our group and others are reporting white matter fMRI activation, specifically in the corpus callosum (e.g., [6-12]). Many of the studies have successfully detected WM activation using interhemispheric tasks to elicit activity in the corpus callosum. Typically, an interhemispheric task involves hemi-field presentation of visual stimuli (light flashes or words and faces) and motor responses from either the ipsilateral or contralateral hand (e.g., [6,11]). In addition to selecting an appropriate task, several other factors appear to enhance sensitivity to WM fMRI activation (e.g., field strength [9]; and MRI sequence [7]).

An important factor that remains to be examined relates to analysis. To-date, the majority of studies have used a hemodynamic response function (HRF) derived from fMRI activation in GM (with the exception of [6,12]). The GM HRF has provided a useful starting point. Analyses using the GM HRF have successfully detected WM activation, with this activation being connected to corresponding GM activation using DTI [10] and experimentally varied as a function of task requirements [8]. As a result, the GM HRF likely provides a reasonable initial approximation for a WM HRF. However, given the known reduction in CBF [4] and CBV [2] and the relative differences in metabolic demand between GM and WM, it is equally reasonable to expect some differences in the WM HRF. Indeed, when applying a model-free approach (finite impulse response [FIR]), Yarkoni and colleagues found a difference in amplitude in addition to a temporal delay when comparing WM HRF to GM HRF [12]. This study applied analyses across multiple data sets, providing the first insight into the WM HRF. However, the most apparent predicted difference of a reduction in the peak response amplitude remains to be tested with the context of a single experimental result.

### The current study

Accordingly, the objective of the current study was to characterize the WM HRF. We used a previously established interhemispheric task that presents word and face stimuli to the visual hemifields and requires a contralateral motor response [6,9]. This task was selected for maximum WM fMRI activation in the isthmus of the corpus callosum. We also used a previously tested asymmetric spin echo (ASE) spiral acquisition method at 4 T [13], which combines T2*- and T2- weighted images for maximum sensitivity to WM fMRI activation [7]. As in prior studies, WM fMRI results were confirmed for both the group and individual levels.

**Figure 1:**
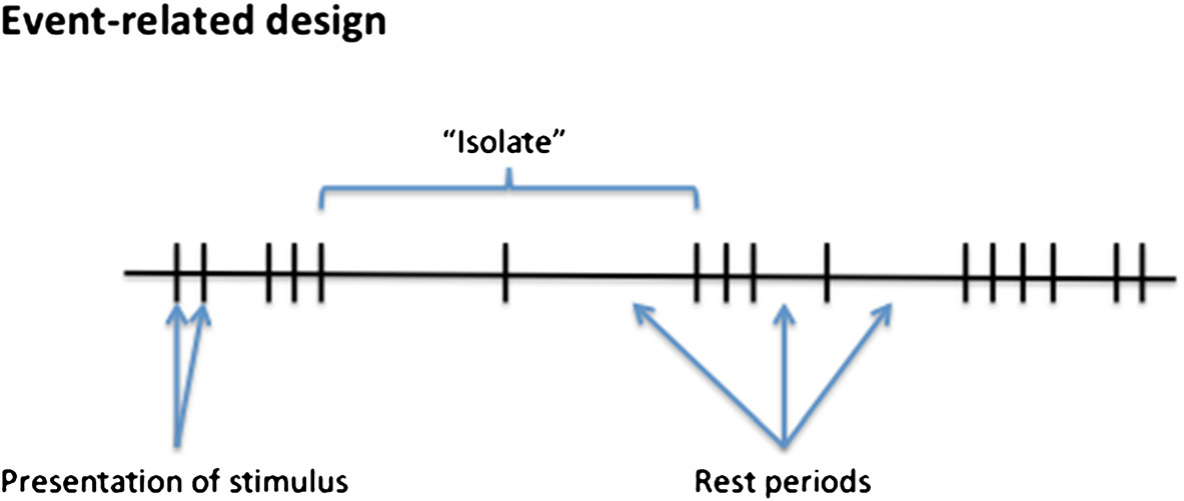
Illustration of the mixed fast/slow event-related design.

To characterize HRFs, we employed a mixed fast/slow event-related design to examine both overlapping and isolated events. Fast, temporally-jittered events were combined with isolated events, in which single events were flanked by rest periods (Figure 1). This design allowed for the generation of activation maps and then the extraction of HRF data from the isolated events (or “isolates”). Two separate analyses were conducted. First, isolates were extracted from the raw data and signal averaged to estimate the WM HRF. The primary purpose of this analysis was to verify the specific response characteristics of the WM activation. Second, similar to the approach taken by Yarkoni et al. the time course data were characterized using FIR modeling [12]. The FIR analysis allowed for the inclusion of all trials in order to better evaluate response amplitude. The main difference in our approach was that we chose to restrict the analysis to WM activation clusters that are robustly detected using the GM HRF model. While this may have restricted overall sensitivity, it provided a specific examination of the HRF for well-established WM activation clusters.

### Study hypotheses

We hypothesized that the WM hemodynamic response characteristics would be similar to those of the GM HRF, given that prior studies have successfully detected WM activation using the GM HRF. However, we also predicted that peak magnitude of the WM HRFs would be reduced relative to the GM HRFs.

## Results

### Behavioural results

Analysis of behavioural data demonstrated that participants performed the Sperry task with a mean accuracy of 80.1%. This level of performance was consistent with previous accuracy results reported from similar interhemispheric tasks [8].

### Group activation results

**Figure 2:**
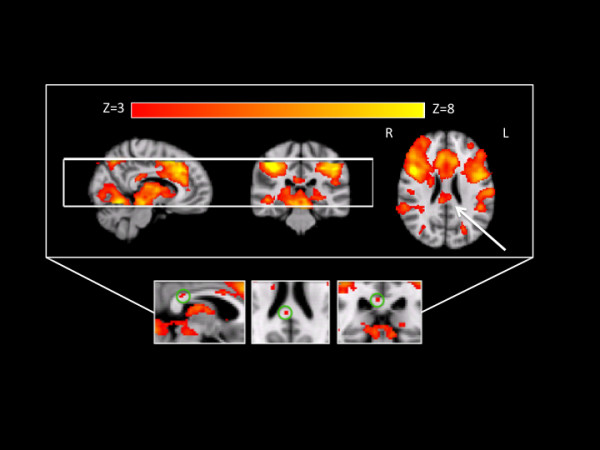
**Group level data showing significant activation in the posterior corpus callosum (arrow) and corresponding GM activation (including both cingulate and parietal activation).** Activation results are presented as Z-scores (Z > 3.0, corrected) using radiological format. The white lines indicate the location of the imaging slab. Inset shows group level data processed without smoothing. Green circles identify succinct, robust white matter activation in the corpus callosum (Z > 4.0).

Figure 2 presents the group activation results showing WM activation in the isthmus of the corpus callosum (arrow, Z > 3.0). Corresponding GM activation was consistent with the task demands and included cingulate and parietal activation at the group level. Of the 16 participants, 14 showed white matter activation in the corpus callosum (87.5%). Although the specific location of the activation varied, most participants (9 of 14) showed activation in the isthmus of the corpus callosum (as demonstrated by the group results). In all 14 cases, the HRF data were obtained from the strongest active clusters for each individual.

The inset of Figure 2 shows group WM activation in the isthmus of the corpus callosum after processing *without* smoothing at Z > 4.0. This provides additional validation that the activation clusters were truly in WM tissue, as opposed to a spatial artifact from nearby GM activation.

### HRF Comparison between WM and GM

**Figure 3:**
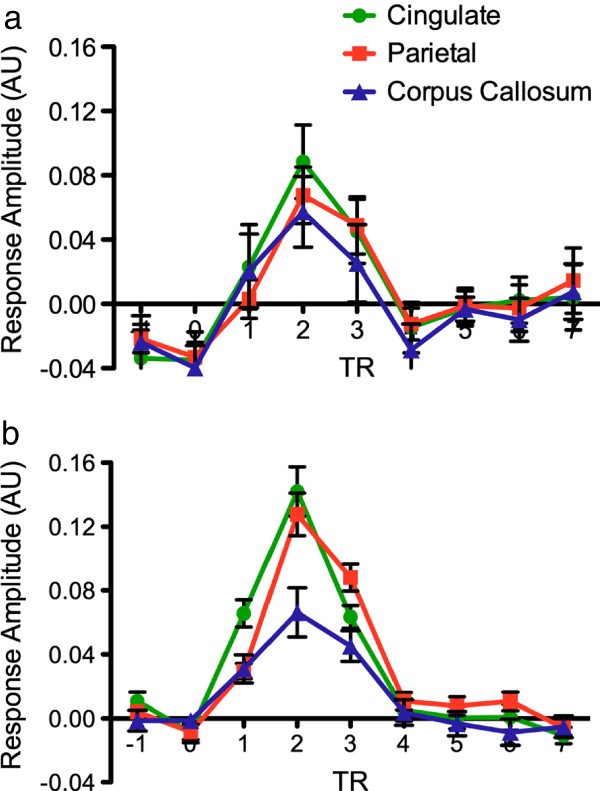
**(a) Hemodynamic response function (HRF) data (arbitrary units; AU) for the raw isolates (n = 8 averaged). **The horizontal axis depicts time in TRs (2 second repetition time). Data were extracted from ROIs in the corpus callosum, cingulate, and parietal areas. (b) HRF data (AU) for the finite impulse response analysis from all three ROIs. All other details as per Figure 3a.

Figure 3 shows the HRFs derived from both the raw (a) and the FIR (b) data. In both cases, the peak response of WM was reduced relative to GM. For raw HRFs (8 trials per subject), there was no significant difference in response amplitude between WM and GM activation (*F* < 1). While the low number of trials resulted in increased response variance, the overall features and temporal characteristics of the WM HRF were similar to those of the GM HRF.

In the FIR analysis (80 trials per individual), there was a significant difference between the peak amplitude for WM HRFs compared to both GM HRFs. The variance in the FIR model was reduced considerably through the utilization of all 80 trials. The repeated measures ANOVA revealed a significant main effect of ROI (GM ROIs > WM ROI) (*F *(2,24) = 13.66, *p* < .0001) and an ROI by time point interaction (*F*(4,48) = 15.7, *p* < .0001), with the largest difference at the HRF response peak. While the WM HRF peak amplitude was reduced, the temporal characteristics and overall features were similar to both the GM HRFs (and the raw WM HRF).

## Discussion

The present fMRI study aimed to compare the WM HRF to GM HRFs. As predicted, while the WM HRF general features and temporal characteristics were similar, the response amplitude was reduced relative to the GM HRFs. While this drop appears to be consistent with known reductions in the ratio of CBF [4] and CBV [2] between WM and GM, it may not reflect a direct relationship between the two factors. Rather the result represents the initial step to evaluate the WM HRF, with more work needed to fully characterize the factors that contribute to response reductions.

Interestingly though, the WM HRF could be detected successfully in the raw time course data even with a lower number of trials. While the increased response variability limited the sensitivity to amplitude differences, the overall trend and temporal characteristics were consistent with the more powerful FIR results. Detecting the raw WM HRF is important because it demonstrated that, similar to GM fMRI activation, WM fMRI activation is observable even within the raw time course data. Given that the analysis focused on ROIs with the strongest activation, the variance is likely also large in this instance. It is likely that increasing both the number of trials and the extent of activation included in the analysis would improve the ability to characterize differences between WM and GM HRFs.

Both the WM and GM activation results closely replicate prior findings on WM fMRI activation [6,8-10]. Similar to these results, it was possible to demonstrate WM activation at both the group level and in the majority of individuals (87.5%). As shown in the group activation maps, most participants showed activation in the isthmus of the corpus callosum. Mazerolle and colleagues showed that white matter fibre tracts connect from the parietal lobes through the isthmus [10]. This network supports the integration of high-level sensory information, such as that required by interhemispheric word and face transfer in the Sperry task [14,15].

It is noteworthy that our results differ from those of Yarkoni et al. [12]. In addition to a reduction in the response amplitude of the WM HRF, this study reported a delay in WM HRF peak latency. The major difference being that Yarkoni et al. [12] did not restrict the analysis to activation localized first using the GM HRF. Taken together, the overall results suggest that an amplitude reduction should be expected, but more work is needed to fully characterize the response timing.

The current results should be interpreted with at least two major caveats. First, in order to identify WM activation, we first used the basic HRF function derived from GM activation. While this approach was necessitated as an initial exploratory step, the GM approximation may not have been sensitive to all WM activation. Therefore, the current results may improve sensitivity to WM activation through response feature optimization, but they do not address whether other WM HRFs exist that do not match the GM HRF. For this, a model-free analysis, like independent components analysis, would provide important additional insight [6,16]. Second, both the temporal resolution and the regional variance in WM HRF time course data remain key factors for future investigations. Future studies should increase the temporal resolution to allow for better characterization of the WM HRF (using reduced but focused spatial coverage). Also, the vasculature supplying the corpus callosum differs from that of other WM tissue; consequently influencing the ability to generalize the current findings.

## Conclusions

Evidence is growing to show that WM fMRI activation is detectable. In the current paper, we replicated WM fMRI activation at the group and individual levels. We then extracted the HRFs from WM and GM ROIs and demonstrated similar features and temporal characteristics. The WM HRF differed only in terms of reduced peak response amplitude. This result is consistent with the greater challenges in detecting WM fMRI activation, but demonstrated that it is indeed possible. Understanding the unique characteristics of WM fMRI activation further improves analysis sensitivity. Identifying active WM tracts has the potential to play a critical role in characterizing network connectivity. Moreover, WM function/dysfunction is increasingly being implicated in a host of brain disorders and diseases.

## Methods

### Participants

Twenty participants (10 females) took part in the study. The mean age of the participants was 23.1 years (age range 19–30). Nineteen of the 20 participants were right-handed. Four of the 20 participants had to be excluded from the analyses due to excessive movement and technical scanner issues. The study had research ethics board approval (National Research Council Research Ethics Board, No. 2006–03 and the Capital Health Research Ethics Board, No. 2006–173) and participants provided informed consent prior to participation.

### Experimental design

The participants performed a modified Sperry task [9,10]. Specifically, we selected the “crossed” conditions, which have been shown to elicit maximum corpus callosum activation [11]. Face and word stimuli were presented to both the right and left hemispheres in order to elicit interhemispheric transfer. Stimuli were either intact or scrambled (i.e., scrambled faces and pseudo-words). Participants were asked to evaluate whether they saw an intact face, scrambled face, intact word, and scrambled word using an MR compatible response pad (four-button, forced choice). Response hand was also crossed for all trials (left hand for words and right hand for faces). Participants were asked to fixate on a point (“+”) that was continually displayed at the center of the screen for the duration of the experiment. All stimuli were presented laterally (>2.3° from fixation) and rapidly (100 ms) in order to initially stimulate only one hemisphere and avoid saccades.

E-Prime (Psychology Software Tools, Inc.) was used to present stimuli, which were displayed using back-projection to a screen mounted inside the magnet bore, and viewed through a mirror mounted on the head coil. Prior to the experiment, each participant performed a short practice task (with feedback) outside of the MRI scanner to ensure complete understanding of the task.

Figure 1 provides an overview of the design structure. We used a mixed fast/slow event-related design to examine both overlapping and isolated events. To minimize subject fatigue, the experiment was divided into two equal sessions (3 minutes and 48 s per session). In total across the two sessions, 80 stimuli were presented with 8 isolated events. Pilot study testing revealed that this design was optimal and further repetitions resulted in response reduction due to habituation (i.e., a confounding factor).

Stimuli were presented in blocks of 1–4 rapidly presented images (100 ms duration, 2 s inter-stimulus interval). The time between stimulus blocks was jittered pseudo-randomly (2000 ms, 4000 ms, or 16000 ms). To isolate events, we included four single events flanked by a 16 s rest period, in both of the trials. All 8 “isolates” were used to generate raw HRFs and all 80 events were used to estimate the finite impulse response (FIR; [17,18]).

### MRI acquisition

Data were acquired from a 4 T Varian INOVA whole body MRI system. Gradients were provided by a body coil (Tesla Engineering Ltd.) operating at a maximum of 35.5 mT/m at 120 T/m/s, and driven by 950 V amplifiers (PCI). A TEM head coil (Bioengineering Inc.) was employed.

Functional MRI data were obtained using an asymmetric spin echo (ASE) spiral sequence [13]. The ASE spiral sequence collects three images per slice per volume (maintaining equal BOLD contrast across the images, but increasing the T_2_ weighting). Seventeen axial slices (4 mm thick, no gap) were prescribed to cover the corpus callosum, as well as the regions extending superiorly and inferiorly. Other parameters for functional imaging were: 64x64 matrix, 220x220mm field of view, 1 shot, TR = 2 s, TR/TE/TE^*^ = 2000/68/27 ms (TE = spin-echo center, TE^*^ = asymmetric echo times). Following the functional MRI, a high resolution ASE spiral (128x128) registration intermediate image and a 3D MP FLASH whole brain anatomical image (72 2 mm axial slices) were collected, with TR/TI/TE = 10/500/5 ms.

### Functional MRI Analyses

In order to increase the overall T2 weighting of the combined image, the three ASE images were combined using an inverted signal weighted averaging algorithm [7].

### Group analysis

Statistical analyses were performed using a model-based approach (General Linear Model) in FMRIB Software Library (FSL) using fMRI expert analysis tool (FEAT) version 5.3 (FMRIB's Software Library). Pre-processing steps included: motion correction using MCFLIRT [19]; brain extraction using BET [20]; spatial smoothing using a Gaussian kernel of FWHM 5 mm; mean-based intensity normalization of all volumes by the same factor; and high pass temporal filtering (0.02 Hz). FILM with local autocorrelation correction was used for the time-series statistical analysis [21]. Z-statistic images were reported using a threshold for clusters determined by Z > 3.0 and a (corrected) cluster significance threshold of P = 0.05 [22]. Images were initially registered to the high-resolution spiral image (3 degrees of freedom; DOF), then to the high-resolution T1-weighted anatomical image (6 DOF) and were finally normalized to standard space (12 DOF) using FLIRT [19,23].

To ensure group-level activation in the WM was not due to spatial smoothing from neighbouring GM activation, a follow-up analysis was performed using the identical approach as outlined above, but without the spatial smoothing (5 mm). Importantly, these results were demonstrated using a high threshold (Z > 4.0) in order to confirm that the activation cluster was localized within WM tissue.

### ROI mask creation

The two fMRI sessions were first concatenated, and motion correction was used to align the images to the first image in the concatenated volume. Activation maps were created from the combined task versus rest condition following the GLM method. From these activation maps, region of interest (ROI) masks were created. To compare activation between white and gray matter, three ROI were selected: corpus callosum (white), parietal lobes (gray), and cingulate cortex (gray). The GM areas were chosen because interhemispheric tasks have consistently been shown to elicit activation in both the parietal lobes and the cingulate cortex [8,15]. For each individual, all activation (Z > 3.0) within the largest cluster was masked for the corpus callosum (with only callosal activation clusters that were fully separated from GM were selected). ROI masks were then created using the strongest activation for both the parietal lobes and the cingulate cortex by matching to the size of the corpus callosum ROI (±5 %). Parietal ROI masks were comprised of both right and left hemisphere activation (each size-matched to the corpus callosum ROI).

### Time course analyses

Both raw signal averaging and FIR analyses were calculated at the individual level to estimate the HRF. The individual HRF estimates were then used to calculate group mean and variance of the time courses.

For the raw signal-averaging estimate of the HRF, the data were first high pass filtered to remove low frequency drifts (0.04 Hz). For each voxel in each ROI, two TRs prior to and eight TRs following each isolate were extracted. These ten TRs (20 seconds) were averaged together for each ROI in all individuals to estimate the pre- and post-stimulus time course (HRF).

FIR analysis was performed using AFNI's 3dDeconvolve program [24]. All stimulus time-points were included to produce voxel-wise estimates of the HRF for eight TRs post-stimulus (16 seconds). A second order polynomial was included in the response model to account for low-frequency fluctuations. The FIR estimate of the HRF was then averaged for each ROI for each individual.

### Statistical analyses

Additional statistical analyses were conducted using a repeated measures Analysis of Variance (ANOVA). These analyses were conducted to compare the peak response amplitudes (3 time points centered around the peak) between the corpus callosum, parietal, and cingulate areas (p < 0.05).

## Authors’ contributions

LMF participated in the design of the study, recruited participants, assisted in the screening of participants, performed the experiment, processed and analyzed data, and drafted the manuscript. MTS provided significant assistance with data processing and analyses. SDB assisted in the screening of potential participants, provided insight and recommendations for the experimental design and analyses, and edited the manuscript. RCND conceived of the study, supervised on the experimental design, assisted with the coordination of all parties involved with the study, and helped to draft the manuscript. All authors read and approved the final draft.
